# Protein trafficking in the mitochondrial intermembrane space: mechanisms and links to human disease

**DOI:** 10.1042/BCJ20160627

**Published:** 2017-07-12

**Authors:** Lisa MacPherson, Kostas Tokatlidis

**Affiliations:** Institute of Molecular, Cell and Systems Biology, College of Medical, Veterinary and Life Sciences, University of Glasgow, Glasgow, U.K.

**Keywords:** mitochondria, oxidative protein folding, protein import, subcellular protein targeting

## Abstract

Mitochondria fulfill a diverse range of functions in cells including oxygen metabolism, homeostasis of inorganic ions and execution of apoptosis. Biogenesis of mitochondria relies on protein import pathways that are ensured by dedicated multiprotein translocase complexes localized in all sub-compartments of these organelles. The key components and pathways involved in protein targeting and assembly have been characterized in great detail over the last three decades. This includes the oxidative folding machinery in the intermembrane space, which contributes to the redox-dependent control of proteostasis. Here, we focus on several components of this system and discuss recent evidence suggesting links to human proteopathy.

## Introduction

It has been estimated that a third of cellular proteins are either integral membrane proteins or have to cross an intracellular membrane to function in an organelle [1]. Hence, these proteins are synthesized by cytosolic ribosomes before being targeted to their correct location. In their unfolded state in the cytosol, the majority of these targeted proteins remain associated with chaperones, which can have wide and variable functions. Most cytosolic chaperones function as either foldases (like the chaperonins, for example, that assist protein folding) or holdases (preventing precursor aggregation). A critical role of some of the cytosolic chaperones (like the Hsp70 and Hsp90 family) is their capacity to interact with receptors located on the surface of organelles via a domain exposed to the cytosol, to ensure proper delivery or preproteins to the correct organelle. In some cases, chaperones may even act as isomerases, oxidases or reductases to reverse incorrect folding and have been shown to be up-regulated in response to environmental stress in order to deal with denatured proteins [2–4].

Mitochondria represent an intriguing case of organelle assembly since the vast majority of their proteins are encoded by the nuclear DNA, and only 13 polypeptides are encoded by mitochondrial DNA (mtDNA) in humans [5]. The cytosolically synthesized mitochondrial proteins must be imported into mitochondria in a process that usually requires cytosolic chaperones, such as Hsp (heat shock protein) 70 and Hsp90. These chaperones prevent the aggregation of precursors by protecting their hydrophobic regions and guide preproteins to the receptors (primarily Tom70 and Tom20) of the translocase of the outer membrane (TOM) complex [6]. Import into specific mitochondrial compartments—the outer membrane (OM), intermembrane space (IMS), inner membrane (IM) or matrix—is determined by targeting signals recognized by various mitochondrial protein import complexes [7]. Most mitochondrial proteins are targeted to the matrix by a cleavable N-terminal presequence. These N-terminal, matrix-targeting signals form amphipathic -α-helices and become proteolytically cleaved after import. Other mitochondrial preproteins may contain internal or C-terminally localized targeting signals, depending on the final destination of the precursor, that dictate the specific import pathway they follow [6].

In the next paragraphs, we will first discuss the salient features of the protein import process across the mitochondrial OM, the IM and the matrix. In this context, we will then dedicate the bulk of our review on the import and oxidative folding in the mitochondrial IMS. The presence of several chaperones and the unique redox control exerted on the import and folding pathways in this mitochondrial sub-compartment have put studying this compartment at the forefront of mitochondrial biogenesis research in the last few years. Concluding this review, we will discuss the intriguing and yet largely unexplored links between the folding and assembly defects in the IMS and human disease.

## Translocation across the mitochondrial OM

**Figure 1.**
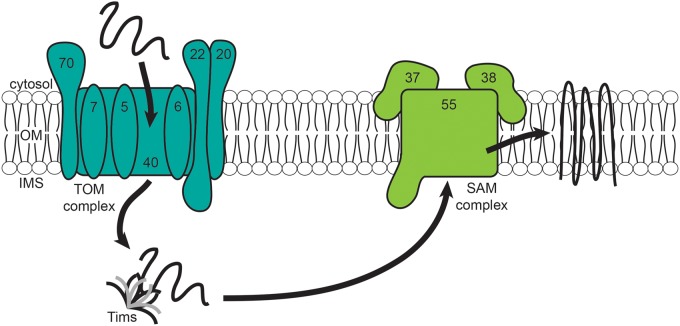
OM protein import pathway. OM precursor proteins are imported via the TOM complex, the general import pore for cytosolically synthesized mitochondrial precursors. Tom20, Tom22 and Tom70 are the receptors of the TOM complex, with the main channel Tom40 and its subunits Tom5, Tom6 and Tom7. The proteins are then chaperoned across the IMS to the SAM complex—with the Sam50 channel and hydrophilic subunits Sam35 and Sam38—by the small Tims.

The TOM complex mediates the import of mitochondrial protein precursors and acts as the general import pore for mitochondrial proteins (Figure 1). Most of the knowledge of the TOM complex comes from *Saccharomyces cerevisiae* (budding yeast) and *Neurospora crassa* (red bread mold), though higher eukaryotic organisms have been shown to contain components homologous to these and with a similar overall structure [1]. The TOM components exposed to the cytosol—Tom20 and Tom70—function as receptors with hydrophilic domains exposed to the cytosol, and are anchored via their N-terminal transmembrane domains. Tom20 and Tom70 recognize N-terminal presequences and hydrophobic precursors with internal targeting sequences, respectively, though they also have some overlap in their function [1,8]. Although Tom20 and Tom70 are the main receptors, there is also Tom71—a paralog of Tom70 that can partially compensate for its function [9,10]—and Tom22, which connects Tom20 to the pore of the TOM complex, and additionally has a large domain in the IMS that helps in the later stages of translocation from the OM to the IM [6,11]. The central component of this pore is Tom40, which forms the central aqueous channel through which precursors enter into mitochondria, and acts as the binding region for precursors [12,13]. In addition to Tom40, there are Tom5, Tom6 and Tom7, which modulate the dynamics and interactions of the import channel. These three subunits are not essential individually, but deletion of all three genes is lethal [6].

The TOM complex is not only crucial for translocation across the OM but is also involved in the insertion of proteins into the OM. However, some OM proteins additionally require other membrane complexes for their import. β-Barrel proteins are guided for insertion into the OM by the conserved β-signal [14]. OM β-barrel proteins lack the cleavable, N-terminal targeting signal that most cytosolically synthesized mitochondrial proteins contain, but instead contain a β-hairpin structure with hydrophobic residues on one side, recognized by Tom20. This β-hairpin has recently been demonstrated as the mitochondrial targeting signal for OM β-barrel proteins, for which the β-signal alone is not sufficient [15].

The SAM complex [sorting and assembly machinery, also called the TOB (topogenesis of OM β-barrel proteins) complex] is involved in the insertion of β-barrel proteins into the OM (Figure 1), such as Porin, Tom40, Sam50, Mdm10 (Mitochondrial Distribution and Morphology 10) or Mmm2 (also known as Mdm34). Sam50 is the main component and highly conserved, containing two domains—the N-terminal hydrophilic region which is exposed to the IMS [containing a POTRA (POlypeptide TRansport-Associated) domain] and the C-terminal domain which forms the β-barrel of the SAM complex. Sam35 and Sam37 make up the two hydrophilic subunits of the SAM complex at its cytosolic surface. Sam35 and Sam50 are essential for viability, and their deletion can block protein insertion into the OM. Sam35 has been shown to recognize the β-signal in a two-step process which opens the Sam50 channel [14]. OM proteins interact with the TOM complex first and move through its pore, while the small Tims bind at the IMS side to the preproteins being translocated and guide them to the SAM complex, which mediates their final insertion into the OM (Figure 1) [6]. Sam37 is vital for the formation of this TOM–SAM complex [16], although it was originally discovered in a temperature-sensitive yeast mutant screen for genes involved in phospholipid metabolism [17].

## Translocation across the mitochondrial inner membrane

**Figure 2.**
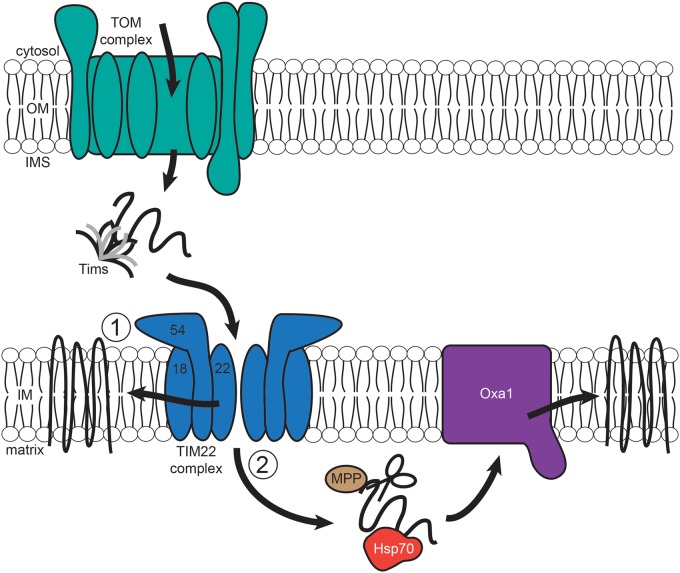
IM protein import pathway. IM precursors are imported into the IMS by the TOM complex, and then chaperoned across the IMS by small Tims to the TIM22 (translocase of the inner membrane 22) complex. The TIM22 complex is comprised of the main channel Tim22 and its accessory subunits Tim18 and Tim54. The proteins then follow one of two routes, either becoming embedded in the IM by TIM22 itself (1), or are released into the matrix where they are cleaved by the action of MPP and Hsp70 before being passed to the Oxa1 (cytochrome oxidase activity 1) complex for membrane insertion.

There are several membrane proteins in the IM, of which some are polytopic and follow the carrier pathway, and others have a single-membrane spanning segment [18]. The TIM22 complex mediates the insertion of carrier proteins [such as AAC (ADP/ATP Carrier) and PiC (Phosphate Carrier)] and membrane-embedded Tims into the IM (Figure 2) [19]. TIM22 requires three protein complexes in order to function—the TOM complex, small Tims to act as chaperones and the TIM22 translocase itself (Figure 2) [20]. The main membrane components of TIM22 are Tim18, Tim22, Tim54 and Sdh3 (succinate dehydrogenase 3). Tim22 and Sdh3 are homologous and can be involved in TIM22 complex assembly on their own [21]. Tim22—related to Tim23 and Tim17—is the central component which is essential for substrate recognition, and forms an insertion channel [22]. Conserved cysteine residues in Tim22 form an intramolecular disulfide bond, which stabilizes Tim22 through its interactions with Tim18 and are vital to the function and stability of the complex [20]. The TIM9/10 complex chaperones preproteins through the IMS to the TIM22 complex by shielding their hydrophobic regions. Tim9, Tim10 and Tim12 also form a complex on the surface of the IM when they are associated with the TIM22 core [23], and Tim54 acts as an adaptor protein in this process by directly interacting with Tim10 [24,25]. Other proteins follow the Oxa1 (cytochrome oxidase activity 1) complex for their insertion into the IM (Figure 2) [26,27], which is a member of a large family of proteins that act to insert proteins into the membranes of mitochondria, chloroplasts and bacteria [6,28,29].

## Translocation into the mitochondrial matrix

**Figure 3.**
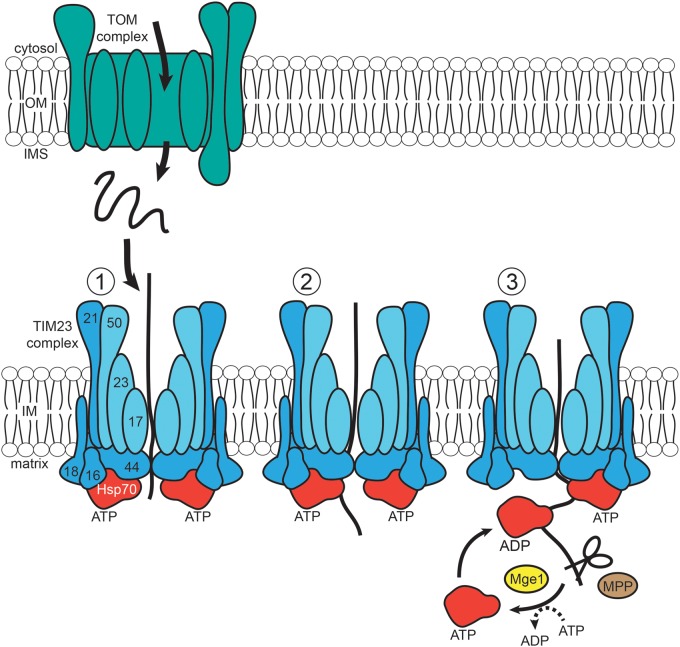
Matrix protein import pathway. Following their import into the IMS by the TOM complex, matrix proteins pass through the IM-embedded TIM23 (translocase of the inner membrane 23) complex, the central junction for the import of matrix proteins. TIM23 is composed of Tim23 and its subunits Tim17 and Tim50, and also interacts with components of the PAM complex (Tim21, Tim44, Pam16 and Pam18) (1). Proteins associate with Hsp70 bound to the matrix side of TIM23 and are translocated across the IM (2). The incoming proteins are cleaved by MPP, and the activity of Hsp70 is regulated by the nucleotide exchange factor Mge1 (3).

The majority (about two-thirds) of mitochondrial proteins are soluble enzymes of the TCA cycle that occurs in the mitochondrial matrix [30]. The dynamic TIM23 complex acts as the presequence translocase of the IM and is a central junction in this import pathway (Figure 3). It recognizes and imports proteins, and then co-ordinates their interaction with the separate TOM and PAM (presequence translocase-associated motor) complexes to sort preproteins both across the IM (to the matrix) and into it. The TIM23 complex and its associated machineries contain multiple components that were mostly initially identified in yeast, but have been shown to be highly conserved in humans [31]. Some of these components have been shown to have genes encoding multiple homologs, though these do not overlap entirely in their function [32–36]. For example, yeast have a homolog of the essential protein Pam18—known as Mdj2 (mitochondrial DnaJ homolog 2)—which can compensate for the function of Pam18 if overexpressed but in itself is non-essential [37]. The TIM23 complex itself has three main components—Tim23, which acts as the channel, Tim50 and Tim17. Tim23 has an IMS domain that acts as a receptor for presequences [38,39], which Tim50 binds to. Tim50, which is anchored to the IM, also interacts with the incoming preproteins from the TOM complex via its IMS-exposed C-terminal domain [40–42]. Tim17, the final component, is essential for both viability and TIM23-mediated protein import, though its function was originally unknown [43,44]. It has, however, been shown to interact directly with Pam17 of the PAM complex and is required for the sorting of IM proteins [45].

Although membrane potential is the initial driving force for the translocation of proteins across the IM (via TIM23 activation), the PAM complex acts as the second driving mechanism—the translocation motor of IM protein import—and is powered by ATP hydrolysis. The mitochondrial Hsp70 (mtHsp70) chaperone forms the ATP-powered core of PAM and binds to preproteins when in an ATP-bound state [with Mge1 (mitochondrial GrpE 1) acting as a nucleotide exchange factor] (Figure 3) [38,43,44,46]. Several, IM-associated co-chaperones are also needed to co-ordinate the activity of mtHsp70 at the channel exit of TIM23—Pam18 and the essential accessory proteins Pam16 and Tim44 [47]. Pam18 is a J-protein which stabilizes the ATP activity of mtHsp70 [32,48,49], whereas Pam16 controls Pam18 activity and forms a stable heterodimer with it [50–52]. Tim44 acts as a scaffold interacting with the PAM import motor via its N-terminal domain and loads mtHsp70 onto the emerging precursor protein via its C-terminal domain [51]. The translocation function of the PAM complex relies on the rearrangement of both domains of Tim44 [53]. Pam17, a non-essential component, is anchored to the IM via its two helices and exposes both its N- and C-terminal domains to the matrix [47]. Another non-essential component of the PAM complex—Tim21—was shown to interact directly with the TOM complex. Both Pam17 and Tim21 bind to TIM23 and are thought to modulate its activity, as protein translocation into the matrix has been shown to require TIM23 in a PAM-bound state, but free of Tim21 [45,47].

## Chaperones and protein targeting in the mitochondrial IMS

The mitochondrial IMS is thought to be evolutionarily related to the periplasm of bacteria. In agreement with this concept, both compartments harbor machineries for oxidative protein folding. Unlike the IMS, however, the periplasm does not contain ATP and the oxidative folding pathways operating in these compartments are not strictly conserved between the two [54]. The IMS is a reducing environment due to the free diffusion of small molecules, such as glutathione, between the cytosol and the IMS (via Porin), whereas the periplasm is an oxidizing environment utilizing the Dsb (disulfide bond) family of thiol oxidases. Despite containing ATP, the IMS is similar to the bacterial periplasm in that it uses ATP-independent chaperones in oxidative folding, and, currently, no ATP-dependent mitochondrial chaperones are known to exist in the IMS [55].

One example of ATP-independent mitochondrial chaperones are the small Tim family—Tim8, Tim9, Tim10, Tim12 and Tim13—which contain unique twin CX3C motifs vital for maintaining their tertiary structure [56]. This motif consists of two cysteine residues separated by three amino acids [57], with each CX3C motif having a variable, 11–16 amino acids between them. The small Tims do not exist in prokaryotes, but are conserved from yeast to higher eukaryotes [58]. Our discussion will focus mainly on the small Tim proteins found in *S. cerevisiae* as these have been studied in most detail, but equivalent proteins are present in human cells. Small Tims act as translocase complexes mediating the import of polytopic proteins (containing multiple helices) of the IM and OM that do not contain a presequence. They are vital in maintaining these hydrophobic substrates in an import-ready state in the aqueous milieu between the two membranes [56]. In yeast, small Tims form two, distinct 70 kDa hetero-oligomeric complexes that act as chaperones of IM proteins—including the mitochondrial carrier family and the membrane-embedded Tims (Tim17, Tim22 and Tim23) [59–61]. These chaperone complexes—known as TIM9/10 and TIM8/13—work in coordination with the IM-embedded TIM22 complex or the OM-embedded SAM complex [2,56]. The subunits of the TIM9/10 complex—Tim9 and Tim10—are encoded by essential genes and exist in excess when compared with TIM8/13 and TIM22 in yeast. The genes encoding the subunits Tim8 and Tim13 of the Tim8/13 complex in *S. cerevisiae* are not essential for cell survival, while, in contrast, the Tim22 gene is essential [62,63]. The TIM9/10 complex binds to the transmembrane segments of precursor proteins as they emerge from the TOM complex, facilitating their passage across the IMS. TIM9/10 complex formation depends on the redox state of its components, with individual Tim9 and Ti­m10 proteins remaining unfolded when reduced [56]. The first, low-resolution 3D structure of the TIM9/10 complex was generated in 2004 while the crystal structures of TIM9/10 and TIM8/13 have confirmed that they function as hexameric complexes composed of three protomers of each of the two subunits [23,63]. This is similar to the structure of Skp (seventeen kDa protein), a bacterial chaperone, which has several, jellyfish-like helical ‘tentacles’ extending from a central structure [23,62,64–66].

Although some bacterial chaperones have been shown to have either a similar function or structure to the yeast small Tim complexes, they remain quite distinct. Despite a common binding specificity for positively charged peptides with aromatic residues, the bacterial chaperone SurA (survival protein A) has a different structure to the TIM9/10 complex. In terms of its function, it has been shown that SurA is not similar enough to replace the role of TIM9/10 in yeast cells, highlighting the difference between them [54].

**Figure 4.**
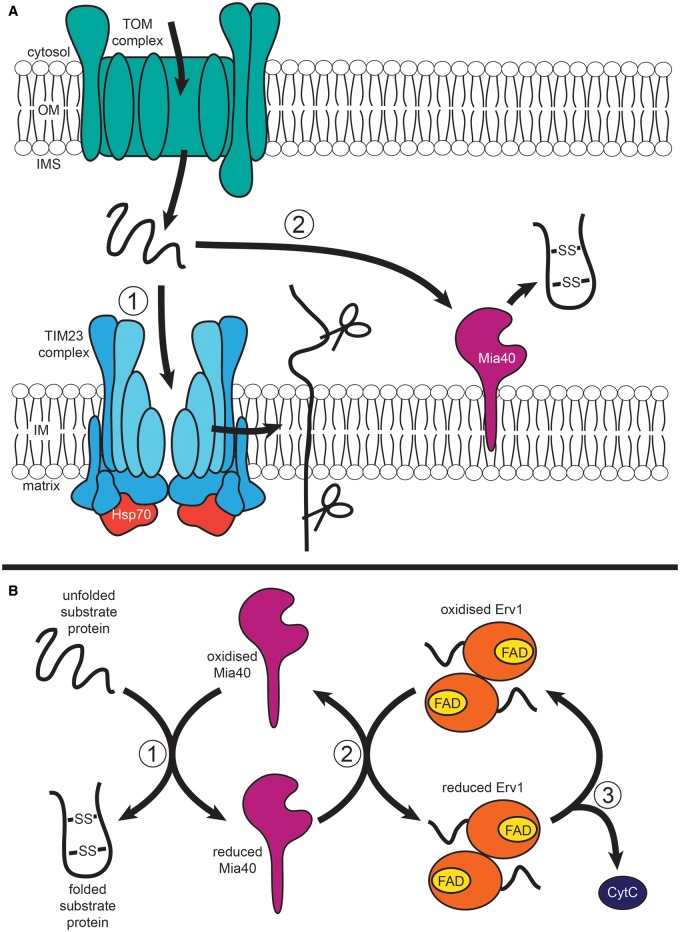
IMS protein import pathway. Precursors are imported into the IMS via the TOM complex, where they then follow one of two routes. (**A**) The stop-transfer pathway of TIM23 (translocase of the inner membrane 23) embeds proteins in the IM before they are cleaved and subsequently released into the IMS (1). Mia40 of the MIA pathway traps proteins in the IMS by introducing disulfide bonds which induce folding (2). (**B**) Unfolded substrate proteins enter the IMS and are folded by Mia40, which causes Mia40 to become reduced (1). Mia40 is then reoxidized by the homodimer Erv1, containing FAD as a cofactor (2). Reduced Erv1 is then reoxidized through the movement of electrons to the final electron acceptor cytochrome *C* (CytC) (3).

The IMS proteins generally follow one of two main targeting pathways to reach their location inside mitochondria. The first one is a variation of the matrix-targeting pathway, called the stop-transfer pathway. IMS proteins that follow this pathway are synthesized with a bipartite N-terminal presequence. The first part of the presequence is a typical matrix-targeting signal that guides the preprotein through the TIM23 complex (Figure 4A) towards the matrix and gets cleaved by the mitochondrial processing peptidase (MPP). The second part of the presequence is a very hydrophobic signal (‘sorting’ or ‘stop-transfer’ signal) that stalls the precursor in transit within the Tim23 inner membrane channel. This stop-transfer signal is then cleaved in an ATP-independent process by the IMS protease IMP (inner membrane protease), which results in the release of the mature protein to the IMS [67]. Recent studies identified Mgr2 (mitochondrial genome required 2), a small, hydrophobic protein, as a key protein in this sorting pathway. Mgr2 interacts with preproteins travelling through TIM23 by recognizing the positive residues of matrix-targeting signal and functions as a checkpoint for the release of preproteins into the IMS, preventing proteins with incorrect targeting signals from being sorted into the IM [68].

The second major targeting pathway for the IMS concerns proteins with conserved cysteine motifs (like the small Tim chaperones), which are critically important for import.

As these proteins lack a typical presequence, they do not engage with the TIM23 complex. Instead, they follow the mitochondrial IMS assembly (MIA) pathway, which introduces disulfide bonds to the imported IMS prepoteins. In this pathway, protein folding is used as a trapping mechanism to retain the folded proteins in the IMS (Figure 4B) [67]. The most extensively studied substrates of this pathway are preproteins containing the twin CX3C motifs (small Tims) or twin CX9C motifs, which, similar to the small Tims, contain two cysteine residues separated by nine amino acids [69]. CX9C protein substrates include proteins involved in cytochrome oxidase assembly [such as Pet191 (petite colonies 191), Cmc1 (CX9C mitochondrial protein necessary for full assembly of cytochrome *C* oxidase), Cox11 (cytochrome *C* oxidase 11), Cox12, Cox17, Cox19 and Cox23] and have been shown to make up 59 proteins of the yeast genome, of which 14 localize to mitochondria [69–71]. Mia40, an oxidoreductase which acts as the disulfide donor protein for imported MIA substrates, also contains a CX9C motif [69]. Some substrates of the MIA pathway, however, do not contain these CXnC motifs but instead have other disulfide bonds—such as Tim17 and Tim22. Tim22 is acted upon by the Mia40, whereas Tim17 is a substrate of Erv1 (essential for respiration and viability 1) [20,72–74].

## Mechanism of oxidative protein folding in the IMS

Mia40 is conserved in eukaryotes but not in prokaryotes [75], and is inserted into the IM by the TIM23 complex that acts on its N-terminal presequence. Mia40 has three disulfide bonds and is bound to the IM by its non-cleavable N-terminus, whereas the core C-terminus domain is exposed to the IMS and interacts with preproteins [76]. These substrates interact with the conserved CPC motif of Mia40, which acts as a lid covering its core, containing a hydrophobic binding cleft stabilized by the disulfide bonds of Mia40's CX9C motif [77]. This allows for the formation of a mixed disulfide intermediate with the substrate protein [78], which is carried out in two steps. The initial, ‘sliding’ step allows for the correct orientation of the substrate with the binding cleft of Mia40, via the non-covalent interaction of hydrophobic residues from the substrate signal helix with the aliphatic side chains of the cleft. The second step, ‘docking’ of the substrate, involves covalent binding of the substrate cysteines to the cysteines of Mia40's CPC active site [79]. This interaction with Mia40 results in the transfer of electrons via a series of reactions that covalently modify the interacting protein. Mia40 itself is then recycled by the second component of the MIA pathway, the flavin adenine dinucleotide (FAD)-linked sulfhydryl oxidase Erv1 (Figure 4B) [67]. Erv1 itself has no structural similarity to other Mia40 substrates, though it is related to the Erv2 of the endoplasmic reticulum and shares a 30% similarity with its C-terminal domain (though their N-terminal domains are different) [80–82]. Erv1 exists as a head-to-tail homodimer [83], and has a human homolog, ALR (augmenter of liver regeneration) [84]. The N-terminus of Erv1 contains an amphipathic helix with flexible loops on either side [85], and is thought to be required for Erv1 dimerization, which is essential for its function. This is shown in plants, where Erv1 dimerizes via its C-terminal cysteine residues as it lacks N-terminal cysteine residues [86,87]. The C-terminus of yeast Erv1 consists of a 4-helix structure which interacts with the isoalloxazine ring of FAD, and forms a platform for the N-terminal domain during electron transfer mediated by conserved pairs of cysteine residues [85]. There are three such cysteine pairs found in Erv1—C30/C33, C130/C133 and C159/C176. The first, N-terminal pair acts as the shuttle disulfide which interacts with Mia40 to transfer electrons to the second pair and then to FAD. The C130/C133 pair co-ordinates non-covalently bound FAD in yeast Erv1 as a core redox center structure, with the third pair acting as a structural disulfide [69]. Erv1 recycles Mia40 via electron transfer from the reduced CPC motif of Mia40 to the N-terminal shuttle cysteine pair of Erv1. Hydrophobic interactions hold the shuttle disulfide in place over the cleft, as with other interacting substrates of Mia40 [88,89]. This electron transfer chain ends with either cytochrome *C* or molecular oxygen as the final electron acceptors [76,78].

## Oxidation-independent, chaperone-assisted protein–protein interactions in the IMS

Although Mia40 has a role in protein import, in recent years it has been appreciated that the key role of Mia40 in the IMS is to act as a chaperone. It was first proposed that Mia40 induced the folding of the α-helical structure of the internal targeting signal recognized by the MIA pathway, demonstrating that internal targeting signals may act as a hub for folding in specific compartments [90]. Both Mia40 and the small Tim chaperones function independently of ATP in the IMS [55] fulfilling the requirement for chaperone activity in this compartment. They both bind transiently to in-transit proteins which have yet to adopt their final conformation. Mia40, however, chaperones soluble proteins that need to be folded in the IMS, whereas the small Tims bind to preproteins of the IM or OM in a similar manner to periplasmic bacterial chaperones [2]. The interacting structures of Mia40 with its substrates are known [90,91]. This is not the case for the small Tim chaperones, for which the structural basis of interaction with substrates has been predicted but not fully confirmed experimentally [54]. Mia40 was shown to act as a chaperone for Atp23, a substrate with 10 cysteine residues, via its hydrophobic binding cleft. Unlike with other Mia40 substrates, import of Atp23 occurred independently of oxidation of these cysteines, as a mutant variant with all cysteines replaced with serines was still able to be imported by Mia40 [92]. Another study of the interaction of Mia40 with Cox17 [91] ­showed that it interacts with the hydrophobic regions of the substrate, which are exposed in unfolded proteins or protein chains with incorrect disulfide bonding. This interaction is dynamic, allowing for Mia40 to bind and release substrates quickly without obstructing the activity of Mia40 with incorrectly folded proteins [91]. Several years ago, it was proposed that the function of Mia40 can be explained by a ‘docking-sliding’ sequential event mechanism [79], as mentioned previously. According to this model, the chaperone function of Mia40 precedes its oxidative capacity by assisting the folding of the incoming precursor in the ‘sliding’ step. Oxidation would then follow in the ‘docking’ step. More recent studies lended independent support for this model: when the Mia40 CPC motif was blocked by the oxidant diamide, Mia40 could still function as a chaperone [93]. Taken together, these studies support the fact that hydrophobic binding is key to the critical function of Mia40 as chaperone (‘holdase’) and an import receptor in the IMS.

## Folding and assembly defects in the IMS system underpin human disease

The first human disease directly associated with a mitochondrial import defect was the neurodegenerative deafness and dystonia syndrome [93], which is caused by a defect in the assembly of the human Tim8 protein (a member of the small Tims family that are substrates of the MIA pathway). A single-point mutation of C66 to tryptophan abolished the capacity of the protein to properly get oxidized keeping it reduced and, as a consequence, inhibited the assembly to its complex with human Tim13 [94]. Defect of assembly of the Tim8–Tim13 chaperone complex caused a multiplicity of mitochondrial defects and was proposed to be the causative effect for the patients suffering from the deafness and dystonia syndrome.

This was the first clear evidence for the importance of the chaperone effects in the IMS and human disease and, in particular, the putative link of the oxidative folding pathway to several human disease states. Indeed, both of the key players in the oxidative folding pathway, i.e. Mia40 and Erv1, have been related to human disorders. The human homolog of Erv1, ALR, has been found to be mutated in a case of three siblings suffering from serious developmental delay, hearing loss, progressive muscular hypotonia and congenital cataract [95]. Specifically, a point mutation in ALR resulting in an exchange of Arginine 194 to His was identified as the cause for this pathological condition. Subsequent biochemical analysis showed that the effect of the mutation at the molecular level was a markedly diminished capacity of the mutant to bind the essential cofactor FAD, with a subsequent loss of stability of the protein, although the catalytic capacity of the mutated enzyme remained largely intact. On the other hand, the human homolog of Mia40 itself (called CHCHD4, coiled-coil-helix-coiled-coil-helix domain containing 4) was shown to have a different expression pattern in tumor cells and to affect the levels of several IMS proteins [96,97]. Knockdown of CHCHD4 correlated with reduced tumor progression and was linked to the stability of HIF1A (hypoxia-inducible factor 1 alpha subunit), one of the central players of the HIF pathway response to hypoxia. To date, this report is the only one that supports a direct association between the mitochondrial oxidative folding/disulfide relay system and cancer [97,98]. In the same vein, an independent study suggested that p53, an important tumor control factor that is redox-regulated, is translocated to mitochondria in a CHCHD4-dependent manner [99]. Overexpression of the oxidoreductase was suggested to result in increased levels of p53 in mitochondria, which in turn affected on the maintenance of the integrity of mtDNA. However, the import pathway for p53 in these studies was not firmly established due to lack of specific import experiments for this protein. The function of p53 in mitochondria remains largely unknown, but this unanticipated link with the MIA machinery; if turns out to be true in further studies, it is intriguing and warrants further studies.

Another major human disorder that has been linked to the Mia40 pathway is amyotrophic lateral sclerosis (ALS). The link in this case affected at the molecular level by the IMS-localized SOD1 (superoxide dismutase 1) protein, which is known for many years to be mutated in several patients suffering from ALS [100–102]. The prevailing concept is that at least some of these mutations of SOD1 inhibit the formation of the disulfide bonds that are critical for the folding and function of the enzyme, resulting in aggregation and thereby leading to impairment of mitochondrial function. The relevance of the MIA oxidative folding machinery in this case is that SOD1 import into the IMS relies on the presence of its chaperone CCS1 (copper chaperone for SOD1), which is an import substrate of the MIA pathway. In this case, the co-operation of Mia40 with the MICOS complex was reported to be critical for the efficient targeting of SOD1 in the IMS [103]. Although most studies suggest that CCS1 is critical for the maturation of SOD1, another possibility put forward by Brazil and co-workers is that the glutathione present in the IMS may be critical for the activation of human SOD1 independently of the effect of CCS1 in this process [104,105].

The mitochondrial disulfide system has also been linked to the neurodegenerative Huntington's disease [106,107], which is characterized by unco-ordinated movement of muscles, depression and stress and, in many cases, linked to mutations in the Huntingtin gene. The levels of IMS redox proteins that are substrates for the disulfide relay system were found altered in a mouse model of Huntington's disease. However, the majority of the mitochondrial phenotypes observed depended on the levels of the mutated Huntingtin protein. These studies are intriguing, but they currently lack a clear molecular explanation as to why the levels of the MIA substrates are specifically altered in the Huntington's mouse model.

It will be exciting in the future to clarify at the molecular level the links between the IMS folding pathways and human disease as they are manifested in different cellular pathophysiological contexts.

## Conclusions

This review aimed to provide the context of the different mitochondria biogenesis import systems with an emphasis on the oxidative folding system in the IMS. The players of this IMS import pathway are involved in chaperone-assisted folding and assembly processes within this sub-mitochondrial compartment. An increasing number of independent studies provides evidence for a connection to redox regulation and how this may underpin several human disorders. Further research will aim to broaden our understanding at the level of mechanistic detail of how redox regulation controls fundamental processes of mitochondrial proteostasis. Such studies hold promise for a more detailed understanding of a large group of mitochondria-related human diseases. Future work in this field will affect on substantial advances in our understanding how mitochondria underpin cellular signaling events in normal cell physiology and disease.

## Abbreviations

ALR: Augmenter of Liver Regeneration
ALS: amyotrophic lateral sclerosis
CCS1: copper chaperone for SOD1
CHCHD4: coiled-coil-helix-coiled-coil-helix domain containing 4
CPC: cysteine-proline-cysteine motif
Erv1: essential for respiration and viability 1
FAD: flavin adenine dinucleotide
HIF1A: Hypoxia-inducible factor 1-alpha
Hsp: heat shock protein
IM: inner membrane
IMS: intermembrane space
Mge1: mitochondrial GrpE 1
Mgr2: mitochondrial genome required 2
MIA: mitochondrial IMS assembly
MPP: mitochondrial processing peptidase
mtDNA: mitochondrial DNA
mtHsp70: mitochondrial Hsp70
OM: outer membrane
Oxa1: cytochrome oxidase activity 1
PAM: presequence translocase-associated motor
SAM: sorting and assembly machinery
Sdh3: succinate dehydrogenase 3
SOD1: superoxide dismutase 1
SurA: survival protein A
TOB: topogenesis of OM β-barrel proteins
TOM: translocase of the outer membrane
